# Carrier Modulation Layer-Enhanced Organic Light-Emitting Diodes

**DOI:** 10.3390/molecules200713005

**Published:** 2015-07-17

**Authors:** Jwo-Huei Jou, Sudhir Kumar, Meenu Singh, Yi-Hong Chen, Chung-Chia Chen, Meng-Ting Lee

**Affiliations:** 1 Department of Materials Science and Engineering, National Tsing-Hua University, Hsinchu-30013, Taiwan; E-Mails: sudhirdubey1983@gmail.com (S.K.); meenu9696@gmail.com (M.S.); 2 Advanced Process Research Division, AU Optronics Corporation, Hsinchu-30078, Taiwan; E-Mails: Yihong.YH.Chen@auo.com (Y.-H.C.); Andrew.Chen@auo.com (C.-C.C.); MT.Lee@auo.com (M.-T.L.)

**Keywords:** OLED, carrier modulation layer, efficiency, lifetime, CRI, SRI, chromaticity tuning, color temperature

## Abstract

Organic light-emitting diode (OLED)-based display products have already emerged in the market and their efficiencies and lifetimes are sound at the comparatively low required luminance. To realize OLED for lighting application sooner, higher light quality and better power efficiency at elevated luminance are still demanded. This review reveals the advantages of incorporating a nano-scale carrier modulation layer (CML), also known as a spacer, carrier-regulating layer, or interlayer, among other terms, to tune the chromaticity and color temperature as well as to markedly improve the device efficiency and color rendering index (CRI) for numerous OLED devices. The functions of the CML can be enhanced as multiple layers and blend structures are employed. At proper thickness, the employment of CML enables the device to balance the distribution of carriers in the two emissive zones and achieve high device efficiencies and long operational lifetime while maintaining very high CRI. Moreover, we have also reviewed the effect of using CML on the most significant characteristics of OLEDs, namely: efficiency, luminance, life-time, CRI, SRI, chromaticity, and the color temperature, and see how the thickness tuning and selection of proper CML are crucial to effectively control the OLED device performance.

## 1. Introduction

Organic light-emitting diodes (OLEDs) have emerged as the most favorable alternative to liquid crystal displays (LCDs) in portable display devices like smartphones, smartwatches, digital cameras, MP3/MP4 players, *etc*. This is because they offer numerous disruptive features, such as energy efficient, lightweight, ultra-thin, mercury free, diffuse surface emission, very high color rendering index (CRI), and potentially low cost [1,2,3,4,5,6]. Recently, some large size OLED televisions (TVs) have also commercialized and their efficiencies and lifetimes are sound at the comparatively low required luminance. In 2013, LG had commercialized 55′′ curved OLED TVs. LG also introduced 65′′ and 77′′ ultrahigh definition (UHD) full-color OLED TVs in March 2014 [7,8,9]. Several large display companies, such as Sharp, AU Optronics, BOE Display, Panasonic, Skyworth, Changhong, and Konka are very close to launching similarly large TVs in the near future [8,9,10]. Although a noteworthy advancement has been made in OLED technology, there are still immense challenges to realize the high efficiency and long lifetime at high brightness, especially for illumination applications [11,12,13].

According to the International Energy Agency’s 2006 report, lighting consumes about 20% of total generated electric energy. It accounts for 30% to 40% of total energy consumption in residential buildings, industrial buildings, and offices. Especially in developing countries, a major amount of building light is consumed by energy-inefficient light sources such as incandescent bulbs [14]. Currently highly energy saving and long-lasting lighting sources are in demand to solve the energy crisis. In recent years, both academics and industries have made a considerable effort to devise novel display and lighting techniques like light-emitting diodes (LEDs) and OLEDs [8,15,16,17,18].

Nowadays, OLED has already reached fluorescent tube efficacy [19]. To realize even more efficient OLED devices, a large number of different approaches have been reported, such as thin device layer structures, low charge carrier injection barriers, high charge carriers (hole and electron) mobilities, balanced carrier injection, effective carrier confinement, effective host-to-guest energy transfer, a wider recombination zone, effective exciton generation on host, effective exciton confinement, p-i-n structures, and tandem structures [20,21,22,23,24,25,26,27,28,29,30,31,32,33,34,35,36,37,38,39,40]. Several of these are especially effective in improving device efficiency at high applied luminance. In past years, the employment of a nano carrier modulation layer (CML) has been recognized as being able to regulate the charge carriers into the available wider recombination zones, and hence obtain a higher device efficiency and reduce the efficiency roll-off at high brightness [41,42,43,44,45]. The incorporation of a high triplet energy CML between the emissive layers could effectively lead the carriers to recombine in a wider recombination zone, and exhibit a marked enhancement in brightness [41,46]. Different terminologies have been used by different research groups regarding the employed nano inter-layers, such as CML (as used in the present review article), carrier-regulating layer, spacer, inter-layer, mixed/blend inter-layer, hole modulation layer, and buffer layer [12,14,46].

**Figure 1:**
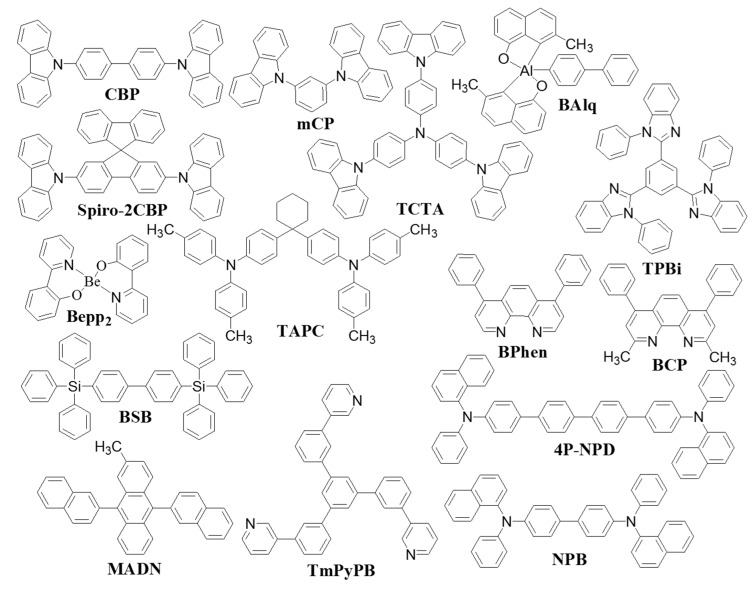
Molecular structures of typical organic materials used as carrier modulation layer.

In past years, different types of materials have been employed as CML (Figure 1), such as ambipolar hosts, electron transporting materials, hole transporting materials, and mixed or blend inter-layer of hole and electron transporting materials. Several research groups have extensively employed numerous types of blend/mixed inter-layer or hybrid spacer, such as 4,4′′-di(triphenylsilyl)-p-terphenyl (BSB):2,7-bis(9-carbazolyl)-9,9-spirobifluorene (Spiro-2CBP) [44], 4, 4′-*N*, *N*′-dicarbazole-biphenyl (CBP):4,7-diphenyl-1,10-phenanthroline (BPhen) [47], CBP:2-methyl-9,10-bis(naphthalen-2-yl)anthracene (MADN) [48], 4,4′,4′-tris(*N*-carbazolyl)-triphenylamine (TCTA):1,3,5-tris[*N*-(phenyl)benzimidazole]benzene (TPBi) [49], TCTA:BPhen, TCTA:bis-(2-methyl-8-quinolinolate)-4-(phenylphenolato)aluminium (BAlq) [50], TCTA:bis[2-(2-hydroxyphenyl)-pyridine] beryllium (Bepp_2_) [51], *N*,*N*′-dicarbazolyl-3,5-benzene (mCP):BPhen, and BH046:BPhen, between the emissive layers to achieve high efficiency, high brightness, very high color rendering index (CRI) and long lifetime OLEDs [44,47,48,49,50,51,52,53,54,55,56]. We have summarized the triplet energy (E_T_), optical energy band gap (E_g_), highest occupied molecular orbitals (HOMO), lowest unoccupied molecular orbitals, hole mobility (μ_h_), and electron mobility (μ_e_) of carrier modulation materials in Table 1. It has been found that the selection of organic molecules for CML were considered according to their properties and suitability.

**Table 1:** Photophysical and electrochemical properties of carrier modulation materials.

Material	E_T_ (eV)	E_g_ (eV)	HOMO (eV)	LUMO (eV)	μ*_h_* cm^2^·V^−1^·s^−1^	μ*_e_* cm^2^·V^−1^·s^−1^	Reference
CBP	2.6	3.5	−6.0	−2.9	2 × 10^−3^	3.0 × 10^−4^	[57,58]
Spiro-2CBP	--	3.38	−5.03	−1.65	1 × 10^−3^	-	[58,59]
mCP	2.9	3.5	−6.1	−2.4	5 × 10^−4^	-	[60]
TCTA	2.79	3.4	−5.7	−2.3	3 × 10^−3^	1.0 × 10^−8^	[57,58,61]
TPBi	2.73	3.5	−6.2	−2.7	-	3.0 × 10^−5^	[62]
BPhen	2.5	3.5	−6.3	−2.9	-	5.2 × 10^−4^	[63,64]
BCP	2.6	3.5	−6.1	−2.6	-	4.6 × 10^−5^	[65,66,67]
BSB	2.76	4.2	−6.5	−2.3	-	-	[44]
Bepp_2_	2.60	3.1	−5.7	−2.6	-	1.0 × 10^−4^	[68]
BAlq	2.18	2.99	−5.57	−2.58	-	3.1 × 10^−5^	[69]
MADN	-	2.90	−5.8	−2.9	-	-	[70]
TAPC	2.87	3.50	5.5	−2.0	1.0 × 10^−3^	-	[71]
4P-NPD	2.3	3.4	5.7	−2.3	6.6 × 10^−4^	-	[72]
NPB	2.3	3.1	5.5	−2.4	8.8 × 10^−4^	-	[73]
PCZAC	2.99	3.35	5.71	−2.36	-	-	[74]

In order to realize high-quality lighting sources, some noteworthy characteristics such as high CRI and high spectrum resemblance index (SRI) are needed. For example, CRI is an important indicator of high-quality illumination, and there are four CRI categories representing different quality, namely very high CRI (>90), high CRI (>75), medium CRI (>40), and low CRI (<40). Very high CRI is crucial for lighting in surgery, photography, museums, *etc.* [12,13]. Moreover, very high CRI with relatively high efficiency has been achieved by the employment of nano CML between two emissive layers with white light complementary emitters; a high-band electroluminescence (EL) spectrum resulted because of effective carrier regulation [12,47,52]. Ironically, a negative CRI value is obtained for some lighting sources. The CRI value should not be less than zero even for the poorest light quality. However, such an inappropriate quantification did and will happen as long as CRI is adopted because of inappropriate artifacts introduced in setting the criteria for CRI calculation, selecting limited and specific samples as standard, and an improper reference light source [75]. To resolve this problem, our group devised a new light-quality index, namely SRI, which quantifies a given lighting source on a lumen rather than power spectrum basis [76]. It has been found that high SRI can be realized by the employment of nano CML between the emissive layers [75,76].

In this article, we have reviewed the effect of incorporating various different types of CML on device performance, e.g., efficiency, maximum luminance, operational lifetime, chromaticity, color temperature, CRI, SRI, and sunlight spectrum resemblance (SSR). Furthermore, this article also describes the effect of nano CML on realizing candlelight-style, sunlight-style, cold- and warm-white, and hybrid white OLEDs. We have discussed the effect of thickness, incorporation position, and functioning mechanism of single and double nano CMLs in a variety of OLED devices.

## 2. Effect of CML on Luminance and Efficiency

The incorporation of CML may effectively lead the carriers to recombine in a wider recombination zone and hence results in a marked luminance and/or efficiency enhancement [41,46]. In 2002, Forrest’s group introduced an exciton blocking, bathocuproine (BCP), nano CML between blue and red emissive layers, and obtained a high efficiency phosphorescent white OLED [77]. This blocking layer is effective in tuning the device chromaticity and achieving a high CRI. Later on, in 2006, they also reported a high efficiency, high luminance, and long lifetime hybrid white OLED by using a CBP nano CML with a thickness larger than the Förster radius (~3 nm). The CBP has effectively minimized singlet-triplet annihilation in the emissive layer, and reduced the exchange energy losses [46]. In 2007, Xie’s group demonstrated efficient hybrid white OLEDs using CBP as nano CML, because it can effectively prevent the Dexter energy exchange between the fluorescent and the phosphorescent emitters [78]. In the same year, Kim’s group employed a 2 nm CML, BPhen, between the blue and red emissive layers in order to prevent a triplet–triplet annihilation (TTA), and realized high efficiency and brightness with stable chromaticity [79]. To reduce the efficiency roll-off and enhance the device performance at high brightness, Leo’s group introduced a TCTA exciton-blocking CML. This TCTA layer could strongly suppress the TTA and hence enhance the external quantum efficiency of the resulting OLEDs [45]. In order to achieve high efficiency and color-stable white emission, in 2008, Ho *et al.* reported a hybrid white OLED by employing a high triplet energy TPBi layer between the fluorescent blue and the phosphorescent orange emissive layers. This 0.8-nm TPBi layer confined both singlet and triplet excitons within the desired emissive zones [80]. In 2012, Jou’s group reported a high brightness and high efficiency deep-blue OLED device with host-free architecture by employing a 1,1-bis[(di-4-tolylamino) phenyl] cyclohexane (TAPC) as a nano CML [41]. In 2013, Wang *et al.* realized an efficacy of 36.1 lm·W^−1^ for pure white OLED by employing a 5-nm electron transporting material BPhen as CML. The high triplet energy BPhen layer has facilitated carrier recombination in the desired emissive layers, and successfully prevented Dexter exchange from the blue emissive layer to the orange counterpart [81]. In 2014, Kim *et al.* also reported a blue phosphorescent OLED by using mCP and TCTA as nano CML. These CMLs improved the efficiency of the device by increasing exciton in a wider recombination zone, and simultaneously reduced efficiency roll-off [82].

Moreover, a few reports also investigated the effect of mixed nano CML on OLED device efficiency and luminance. In 2006, Leo’s group reported a hybrid white OLED using a mixed nano CML e.g., TCTA:TPBi between the fluorescent blue and the phosphorescent green and red emissive layers, which prevents the strong charge carrier imbalance and enables the transport of holes and electrons [50]. In order to balance the charge carriers in desired emissive layers, Kim’s group investigated the effect of different pairs of mixed nano CML such as CBP:BPhen, CBP:MADN, mCP:BPhen, and TCTA:BPhen, to realize high efficiency in hybrid white OLEDs [48,54]. It has also been established that the ratio of mixed CML components has also extensively affected the EL characteristics of hybrid white OLEDs [44]. Mixed CML also suppressed phosphorescent quenching by energy transfer to the low-lying non-radiative triplet state of a blue fluorescent dopant in hybrid white OLEDs [82]. Recently, Jou’s group reported a high efficiency and low color temperature OLED by using BSB:Spiro-2CBP blend interlayer as CML, and a highly efficient candle light-style OLED by using a mixed CML of TCTA:TPBi [47,53]. They also used numerous type of CMLs, such as TCTA, TPBi, BCP, 1,3,5-tri(m-pyrid-3-yl-phenyl)benzene (TmPyPB), BSB, and Spiro-2CBP to realize high efficiency [53,83,84,85] and very high CRI [13,86], warm- and cold-white OLED devices.

**Figure 2:**
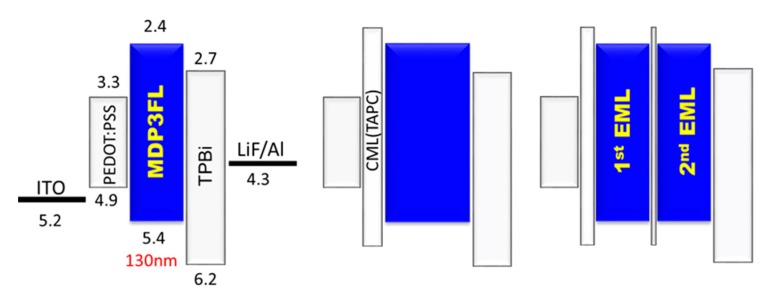
Devices with no CML, single, or double CMLs. (Reproduced from ref. [41]).

A host-free deep blue OLED realized an increment in its maximum luminance from 5250 to 6650 cd·m^−2^ by employing a 5-nm TAPC layer as CML between the hole-transporting layer and the emissive layer. The resulting maximum luminance was further increased from 6650 to 8810 cd·m^−2^ as the CML thickness increased from 5 to 15 nm. Although the maximum luminance can be increased with the increase of CML thickness, the blue emission showed a bathochromic effect, and subsequently the efficacy started to drop. The efficacy dropping may be attributed to the employed CML that confines the injected electrons into the emissive layer due to a large energy barrier of 0.4 eV between the layer of TAPC and 2,7-bis-{2[phenyl(m-tolyl)amino]-9,9-dimethyl-fluorene-7-yl}-9,9-dimethylfluorene (Figure 2) [41].

**Figure 3:**
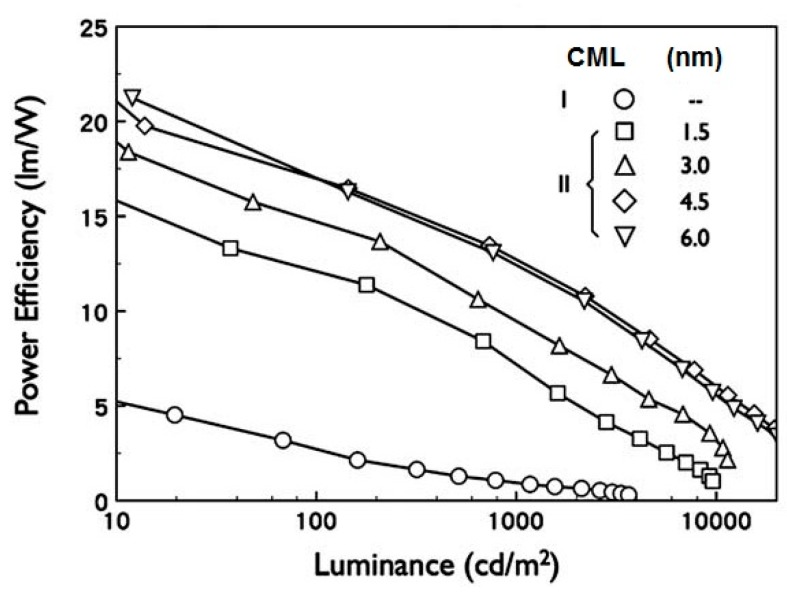
Effect of the different CML thickness on the resulting power efficiency of the white OLED devices (Reproduced from ref. [13]).

In addition, researchers have also investigated the effect of double nano CML on OLED devices, and reported that the two key factors, namely appropriate CML thickness and CML position, played a significant role in enhancing the efficiency and luminance of OLEDs. Jou’s group had reported a double CML OLED in which the first CML thickness was kept at 10 nm and the second CML thickness varied from 5 to 2 nm; the maximum luminance increased from 5960 to 7740 cd·m^−2^ and the external quantum efficiency (EQE) increased from 4.3% to 4.5% at 1000 cd·m^−2^, the same as observed for single CML contained devices [41]. As shown in Figure 3, white OLED performance was notably altered by varying the CML thickness.

**Figure 4:**
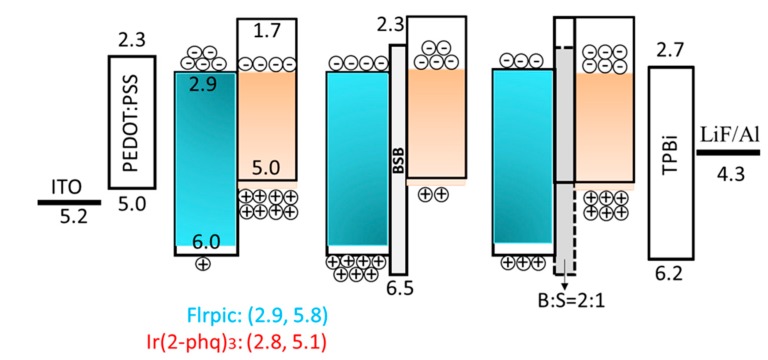
Schematic energy diagram of low color temperature OLEDs without CML and with a neat BSB layer and blend BSB:Spiro-2CBP layer as CML (Reproduced from ref. [53]).

Our group also investigated the thickness effect of TCTA nano CML on hybrid white OLEDs. For example, the device efficiency increased from 7.0 to 13.0 lm·W^−1^, at 1000 cd·m^−2^, as the CML thickness increased from 1.5 to 4.5 nm. However, as the CML thickness further increased to 6 nm, device efficiency decreased to 12.7 lm·W^−1^ because of the large number of electrons blocked from the phosphorescent to fluorescent emissive layer [13]. Jou’s group had demonstrated low-CT phosphorescent OLED devices by employing a blend nano CML of BSB:Spiro-2CBP between the sky blue and the orange red emissive layers. Figure 4 shows the device architecture with corresponding energy levels and the plausible distribution of charge carriers (holes and electrons) in low-CT OLED devices under different conditions of the device architecture [53].

Both the power efficiency and the EQE of the device abruptly dropped, at 1000 cd·m^−2^ for example, from 25.6 to 5.7 lm·W^−1^ and 17.2% to 5.1%, while the color temperature increased from 1860 to 2780 K as a 3-nm BSB layer was introduced between the blue and the orange red emissive layers. As demonstrated in Figure 4, BSB possessed a 0.5 eV barrier to hole injection, and the maximum amount of holes blocked into blue emissive layer. This also explains why the device color temperature increases. However, a 3-nm mixed CML with a 2:1 weight ratio of BSB:Spiro-2CBP in the device markedly improved device performance. The resultant device showed an efficacy of 29.1 lm·W^−1^, and EQE of 20.8%, at 1000 cd·m^−2^, with a CT of 940 K. This noteworthy enhancement resulted because of blend nano CML, which improves the distribution of the charge carriers into the available recombination zones and balances the carrier injection [53].

Furthermore, severe efficiency roll-off is often observed in OLED devices, and is especially unfavorable to their applications for lighting purposes, wherein high efficiency at high luminance is required for energy saving. The efficiency roll-off may arise due to exciton quenching mechanisms such as TTA, singlet–triplet annihilation (STA), guest–guest annihilation, host–guest annihilation, host–host annihilation, all of which also play a crucial role in roll-off phenomena (for efficiency roll-off details, see the review article of Murawski *et al.* [87]). Notably, these mechanisms are associated with the dynamics of injected carriers, *i.e.*, the distribution of charge carrier density in the emissive layer. In order to suppress the efficiency roll-off, numerous approaches have been reported, for example, using light-emitting materials with lower excited state lifetime [4], preventing the aggregation of emitter molecules [58,88,89,90], choosing high mobility carrier-transporting materials [91,92,93], using triplet managers [94,95], and widening the recombination zone [96,97,98,99,100]. In past years, various techniques have been developed to realize a wide recombination zone, such as double emissive layers, a mixed-hosts emissive layer, graded emissive layer architecture, and nano CML between two emissive layers. Among these, nano CML is one of the most favorable techniques, especially for hybrid white OLEDs, to prevent the TTA and broaden the recombination zone. For example, Leo’s group successfully suppressed the efficiency roll-off in a hybrid white OLED by introducing a 2-nm TCTA layer between the fluorescent and phosphorescent emissive layers [45]. Ma’s group reported reduced efficiency roll-off in highly efficient hybrid white OLEDs by employing a 3-nm NPB as a CML, which balances the carriers’ injection and widened the recombination zone. The employment of CML showed effective carrier injection balance and confinement of the triplet/singlet excitons within the emissive layers, resulting in high efficiency [42].

## 3. Effect of CML on Lifetime

White devices have realized nearly 1.5 times higher efficacy than fluorescent tubes, but their lifetime, especially at high brightness, remains a critical issue that limits the commercialization of OLED technology in lighting applications. The operational lifetime (t_50_) of OLED devices is defined as the time in which its luminance decreases to half of its initial value. Therefore, luminance degradation is one of the crucial problems for OLED device lifetime. In recent years, both academics and industries have made several efforts to enhance the operational stability of OLEDs (for a review of operational stability enhancement, see our book chapter [101]). Researchers have reported several approaches for long lifetime devices—for example, design and synthesis of high thermally and electrically stable organic materials, mixed host device architecture, and electrode interfaces (e.g., ITO/HTL or ETL/Al) modifications [101]. Besides these approaches, a nano CML as exciton blocking layer and buffer layer also markedly enhance the operational stability of OLEDs. Here, we describe the effect of nano CML incorporation on the lifetime of monochromatic and white OLED devices. Moreover, extensive improvement in OLED lifetime was observed by the reduction of the Joule heating, which occurred due to the excessive injection of unbalanced carriers [46,102]. In order to obtain OLEDs with long lifetimes as a result of reduced Joule heating, it becomes imperative to extract high luminance and high efficiency at a lower current density [103].

Incorporation of mixed CML between the fluorescent and phosphorescent emissive layers has effectively controlled exciton quenching and singlet–triplet annihilation by limiting the singlet–triplet excitons. For example, Forrest’s group reported the hybrid white OLED, with a nano layer of CBP as CML between the fluorescent blue and the phosphorescent red and green emissive layers, has markedly enhanced the efficiency at high brightness, and extended the device lifetime [46]. Lee’s group investigated the effect of a 5-nm mixed CML, TCTA:PH1, on the lifetime of hybrid white OLEDs and investigated the relationship between the CML compositions and color stability of the hybrid white OELD after lifetime measurement. The chromaticity changes of OELD devices could be diminished by managing the composition of CML. In addition, the lifetime of hybrid OLEDs was also improved in devices as the concentration of PH1 was increased [104]. Besides these methodologies, employment of CML as a buffer layer also improves the efficiency and operational lifetime of OLEDs. In 1996, Tang *et al.* reported a highly stable OLED by using a CML of copper phthalocyanine (CuPc) on the anode (ITO) surface, which improved the charge injection from ITO to HTL and hence achieved a noteworthy enhancement in device stability [105]. A magnesium (Mg) buffer layer between ITO and HIL/HTL layer had effectively blocked the indium ions’ diffusion into the organic layer and resulted in an extensive improvement in device lifetime [106]. Jou’s group had reported the incorporation of double CML within the emissive layer, and successfully increased the maximum luminance of a host-free deep-blue OLED device [41].

The higher obtainable luminance could presumably enable the device to exhibit a longer lifetime since a lower operation voltage would hence be needed, on the basis of the same required luminance, by noting the device lifetime to be inversely proportional to the applied voltage. Liu *et al.* reported a highly efficient hybrid white OLED with extremely long lifetime by using electron-transporting nano CMLs such as Alq_3_, BAlq, TPBi, and Bepp_2_, which successfully prevent Dexter energy transfer from the fluorescent to the phosphorescent emissive layers, manage excitons in a wide recombination zone, and enhance the device performance. The resultant device exhibited an operational lifetime (t_50_) of 76 h at 10,000 cd·m^−2^ initial brightness as a high mobility electron-transporting material; Bepp_2_ is used as a CML [107]. Lee *et al.* reported a blue OLED device with a lifetime (t_50_) of 27,500 h, at 500 cd·m^−2^ initial brightness, by using a multiple quantum well architecture with bipolar mixed CMLs of mCP:TPBi (1:1). The bipolar nature of mixed CML successfully distributes the charge careers in a wide recombination zone, and realized a device lifetime 3.5 times longer than that of the device without the CML counterpart [108].

**Figure 5:**
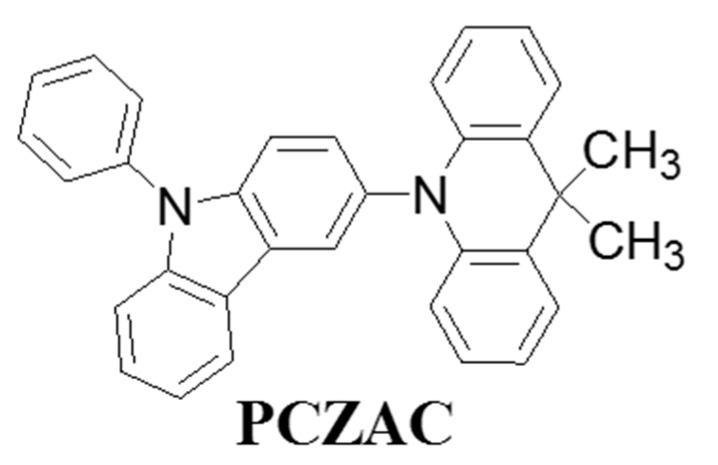
Molecular structure of exciton-blocking material PCZAC as nano CML.

Most recently, Lee’s group designed an acridine derivative, 9,9-dimethyl-10-(9-phenyl-*9H*-carbazol-3-yl)-9,10-dihydroacridine (PCZAC) (Figure 5), for blue OLEDs to realize high device efficiency and longer device operational lifetime. [74] The resultant PCZAC compound possessed high triplet energy of 2.99 eV and high glass transition temperature of 101 °C. When a PCZAC compound was employed as an exciton blocking layer in a blue phosphorescent OLED device, the architecture of ITO/DNTPD/BPBPA/PCZAC/EML (mCBP + 10% Ir(dbi)_3_)/ETL (LG201)/LiF/Al exhibited a lifetime (t_70_) of 180 h at initial luminance of 500 cd·m^−2^, which is over eight times higher than that of control device (ITO/DNTPD/BPBPA/EML/ETL (LG201)/LiF/Al) counterpart.

## 4. Effect of CML on CRI and SRI

A number of approaches have been demonstrated to fabricate OLED with a full wavelength spectrum with a very high CRI [12,109,110,111]. Typically, the fabrication process for high CRI white OLED includes two typical approaches based on the structure of emissive layers. They are multiple emitters within a single emissive layer [12,109,110], and multiple monochromatic emissive layers to achieve broad visible spectrum [111,112]. However, it is complicated to realize a high efficiency OLED device with very high CRI.

**Figure 6:**
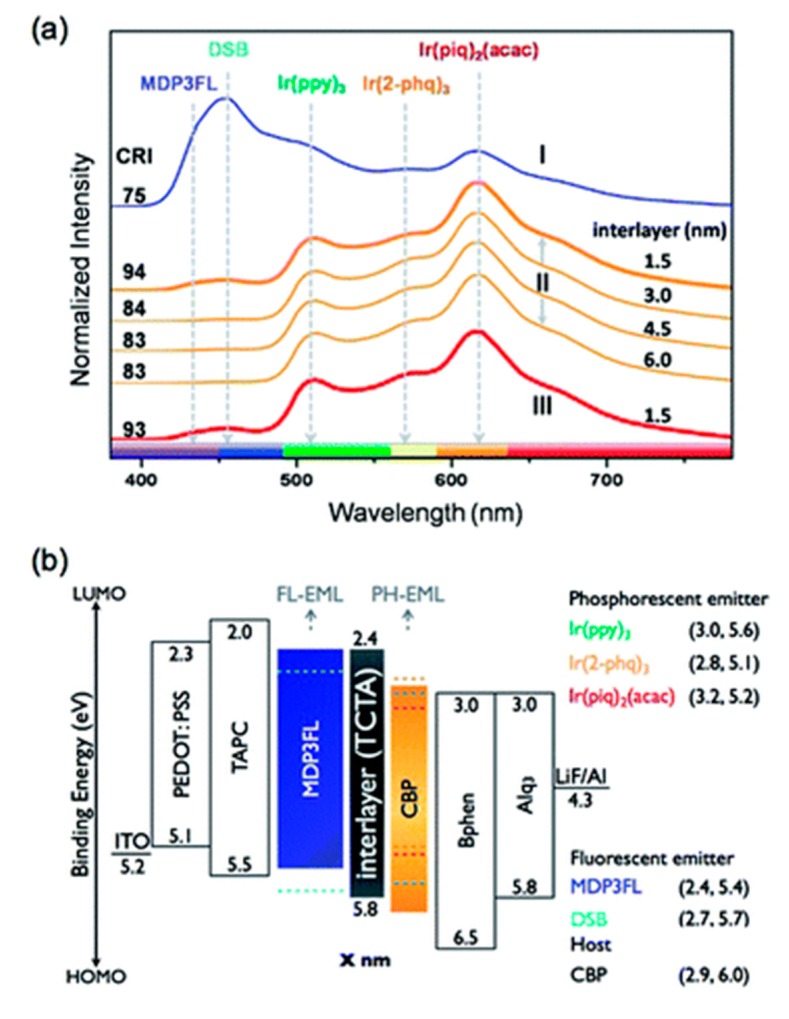
(**a**) EL spectral results of white OLED devices with and without a CML; (**b**) energy diagram of the white OLED devices (Reproduced from ref. [113]).

A nano CML hence plays a significant role in achieving a very high CRI with full-wavelength spectrum OLED by regulating the carrier injection, widening the recombination zone, and controlling the excitons in desired emissive layers. In 2011, Jou’s group reported a very high CRI of 93 for hybrid white OLED by introducing a 1.5 nm TCTA layer as CML [113]. The resultant device shows five bands in the electroluminescent spectrum with an emission wavelength ranging from 400 to 750 nm (Figure 6a).

**Figure 7:**
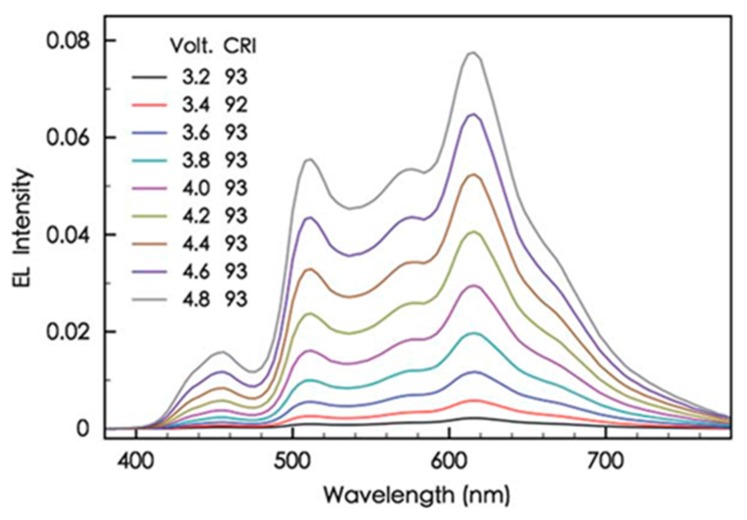
The EL spectra and the corresponding CRI of the OLED device with a CML at applied voltage between 3.2 and 4.8 V (Reproduced from ref. [113]).

Without the nano CML, a device showed a high-intensity major emission in the blue (fluorescent emitter) wavelength region (Figure 6a), while the TCTA nano CML-based device exhibited a strong red emission (phosphorescent emitter) because the majority of holes are blocked by the TCTA carrier-regulating layer possessing a 0.4-eV energy barrier, as shown in Figure 6b. This explains why the lack of a CML device resulted in relatively poor device efficiency. At 1000 cd·m^−2^ for example, the resultant device realized the lowest CRI of 75 and an efficacy of 1 lm·W^−1^, which is extremely poor compared to the device with a 1.5-nm CML counterpart [113]. Unlike typical OLED devices, the device with 1.5 nm CML (TCTA) shows a constantly high CRI of 93 upon increasing the driving voltage from 3.2 to 4.8 V, as shown in Figure 7.

**Figure 8:**
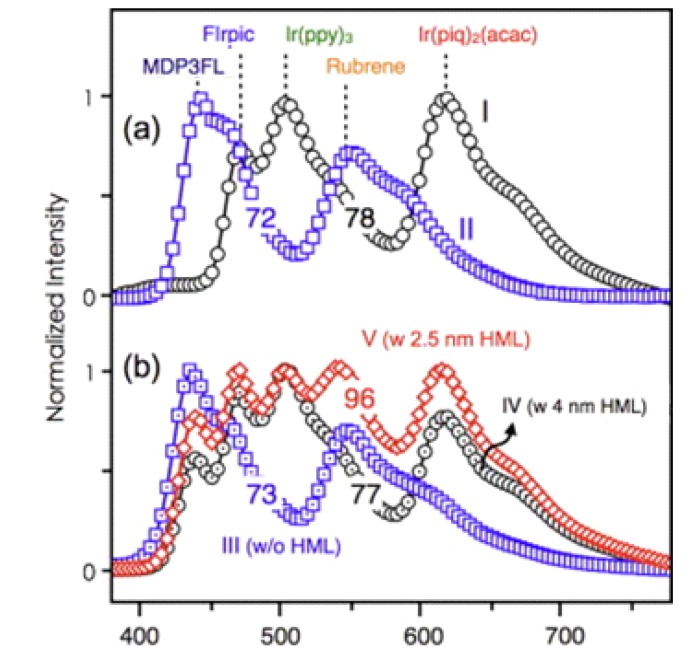
EL spectra of (**a**) single white emissive layer OLEDs and (**b**) the double white emissive layers containing OLEDs (Reproduced from ref. [114]).

Moreover, Jou’s group reported, at 100 cd·m^−2^ for example, a CRI of 98 with an efficacy of 8.3 lm·W^−1^ by employing a 2.5 nm TPBi layer as a CML between the fluorescent and the phosphorescent white emissive layers [114]. As shown in Figure 8, the EL spectra of single emissive layer-based fluorescent and phosphorescent white OLEDs exhibited double peak emission with a CRI of 78 and 72, respectively. When the fluorescent and phosphorescent white emissive layers were stacked in a single device, the resultant CRI remained virtually unchanged. As an optimized 2.5-nm CML was incorporated between these white emissive layers, the CRI value was enhanced to 96. The additional 2.5 nm TPBi layer as CML precisely regulated the carrier injection between the fluorescent and phosphorescent emissive layers [114].

In 2012, Ma *et al.* reported a hybrid white OLED with high CRI of 90 by using a bipolar TCTA:Bepp_2_ as nano CML. This CML improved the charge carrier injection from the electrodes and was responsible for the wide recombination zone to avoid exciton quenching. Subsequently, the nano CML provided a stable broad white spectrum because of its bipolar character [51]. In 2012, Leo’s group also reported a white OLED with a CRI of 82 by using a multiple emissive layer structure with TCTA and SPP01 as nano CMLs. The thickness of CMLs can balance the Forster and Dexter energy transfer between host and guest and prevent non-radiative emission. The CMLs also managed the recombination zone to provide a wide spectrum and high efficiency [115]. In 2013, Linghao *et al.* reported a white OLED based on an excimer Pt compound that achieved a very high CRI by using a mCP as nano CML. A high triplet energy CML accomplished an exothermic energy transfer to guest and confined the excitons in emissive layers. This results in reduced efficiency roll-off and enabled a very high CRI of 97 [116]. In the same year, Jou’s group also reported a physiologically friendly OLED device with high or very high CRI. For example, our group fabricated a chromaticity tunable between dusk-hue and candlelight OLED device with a high CRI of 85 by employing a nano layer of TPBi as CML [84]. In 2014, Sun *et al.* investigated an extremely low efficiency roll-off white OLED with very high CRI of 94 by using a TmPyPB as CML, which effectively prevented Forster energy transfer and retained a sufficient blue emission, an inevitable light component of white emission [117].

It has been found that the conventional CRI is limited because it is unable to provide a direct correlation of any artificial light source with corresponding natural light. In some cases, even a negative CRI value is obtained, e.g., the value for the poorest light quality should be zero instead of an unreasonable negative one, which is not providing truthful information about the light quality. In past years, numerous efforts have been made to realize an appropriate light quality index that can directly provide the quality of light corresponding to natural light at any color temperature. In 2011, we had reported an index, sunlight spectrum resemblance (SSR), to quantify the resemblance of the power spectrum of any given light source with that of sunlight at the same color temperature [83]. Two problems would, however, arise as the index is employed. First, the reference sunlight spectrum is not universal; *i.e.*, the power spectrum of the reference sunlight frequently varies with varying time, weather, and/or latitude, and is not at all smooth, especially in the long wavelength region at low color temperatures. Second, in the index calculation, equal weight is assigned to all different visible lights with different wavelengths, which would lead to an energy-wasting lighting design as an SSR near 100 is pursued. That is because quite a lot of power would be wasted on generating deep blue and deep red regions not visible to the human eye in order to achieve the ultimate quality.

In order to resolve these problems, our group proposed a new light quality index, namely SRI, which quantifies a given lighting source on a lumen rather than a power spectrum basis. It employs the universally obtainable black-body radiation as the reference instead of sunlight spectrum. This luminance-based SRI of a given light source can be calculated on the basis of the same luminance by employing Equation (1) [75,76]:

(1) $$SRI\;=\;\frac{\int^{\text{​}}L(\text{λ},T)dy}{\int^{\text{​}}L_{BR}(\text{λ},T)dy}\;\times \;100\%$$

where *L_BR_*(λ, *T*) is the luminance spectrum of the backbody-radiation and *L*(λ, *T*) is the overlapping area between the luminance spectrum of the studied light source and its corresponding blackbody-radiation.

**Figure 9:**
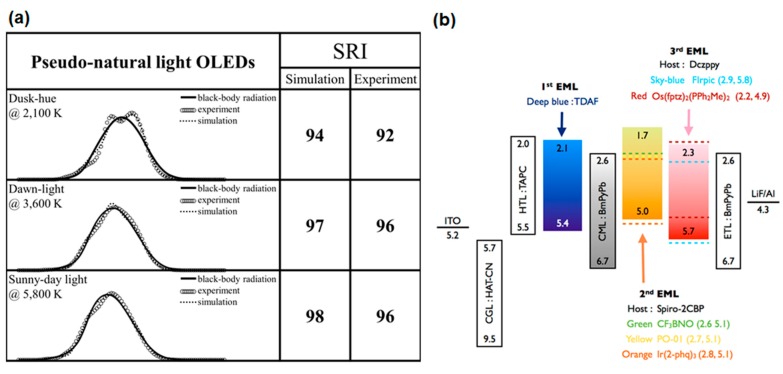
(**a**) The luminous spectra obtained from simulation and experiment for the pseudo-sun light at dusk-hue, dawn, and sunny day (noon) by employing an organic light-emitting diode (OLED) with the six different emitters; (**b**) Device architecture of pseudo-natural style OLED with molecular structure of BmPyPB (Reproduced from the ref. [75]).

Most recently, our group reported a series of pseudo-natural style OLEDs with SRI values ranging from 92 to 96 by dispersing the six emitters into three different emission layers. In order to avoid the singlet–triplet exciton quenching, an electron transporting material, 1,3-bis(3,5-dipyrid-3-yl-phenyl)benzene (BmPyPb), has been employed as a nano CML between the fluorescent deep-blue and the phosphorescent orange-red emissive layers (see Figure 9b for OLED device architecture and molecular structure of BmPyPb) [75].

## 5. Effect of CML on Chromaticity Tuning

High efficiency and chromaticity tunable OLEDs are striking for numerous applications. However, it is difficult to achieve both chromaticity tunability and high efficiency simultaneously. In recent years, several efforts have been made to devise the chromaticity-tunable OLEDs—for example, employment of color temperature tunable emitters, variant dopant concentrations [118], employment of appropriate host materials with suitable work function [119], shifting the ultra-thin yellow emissive layer position in a blue matrix of complementary emitter-based white OLED devices [120], and employment of nano CMLs [76,121]. Among these, the nano CML approach is considered to be the most favorable because of its numerous superlative characteristics, such as carrier regulation function, ability to confine the excitons within the specific emissive layer, and choice of materials for CML. For example, Forrest’s group reported a chromaticity tunable OLED device by inserting a 5-nm BCP layer as an exciton-blocking layer. The CIE coordinates of chromaticity-tunable OLEDs varied from (0.35, 0.36) to (0.37, 0.40) [77]. Xie’s and Li’s groups reported a chromaticity-tunable hybrid OLED device by varying the CML thickness from 0 to 8 nm, where CBP is used as a nano CML. The resultant devices exhibited color coordinates from (0.24, 0.24) to (0.36, 0.40) [78]. Liu *et al.* reported a color-tunable OLED device by using a 16-nm CML between the blue and yellow emissive layers. The resultant device exhibited a current efficiency of 77.4, 70.4, and 33.7 cd·A^−1^, respectively, for blue, white, and yellow emission. Furthermore, they also found that as the CML thickness varied from 8 to 24 nm, the emissive zone shifted towards the blue emissive layer because of the maximum number of holes blocked at the CML/blue emissive layer interface. Resultant chromaticity coordinates ranged from (0.23, 0.32) to (0.38, 0.41) as luminance increased from 1000 to 5800 cd·m^−2^ [122].

**Figure 10:**
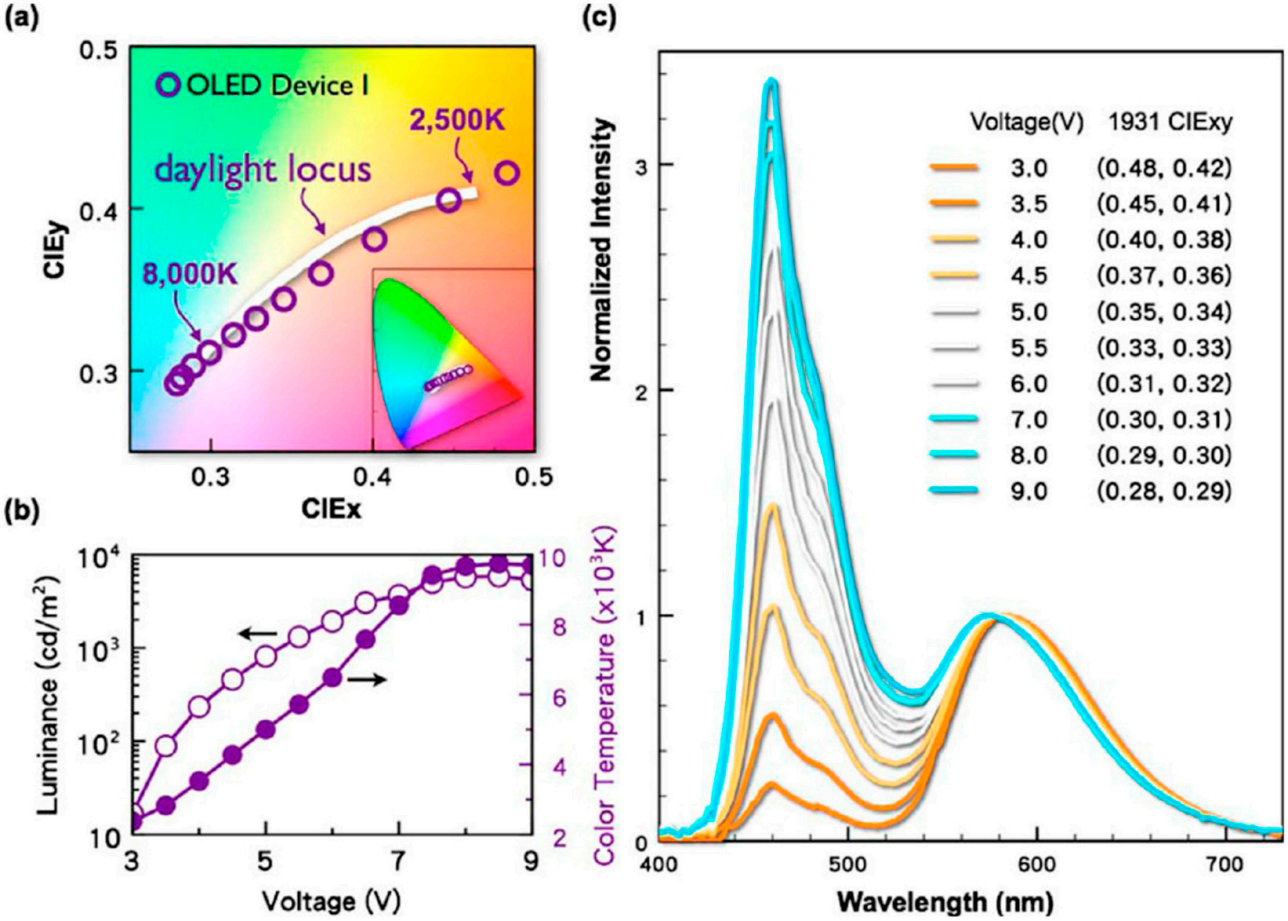
Chromaticity and color-temperature characteristics of the sunlight-style OLED (**a**) emission track on CIE 1931 chromaticity space matches closely to the daylight locus between 2500 and 8000 K; (**b**) color temperature changes from 2300 to 9700 K and brightness from 20 to 5900 cd·m^−2^ as the voltage increased from 3 to 9 V; (**c**) EL-spectra at various applied voltages (Reproduced from ref. [86]).

In 2009, Jou’s group reported a sunlight-style chromaticity-tunable OLED device by employing a 3-nm CML of TPBi between green and red EMLs. This device exhibited an emission track that closely matches with the daylight locus on CIE 1931 space (Figure 10a). As shown in Figure 10c, the EL-spectra of device initially shows a predominantly red-emission spectrum at 3 V with CIE coordinates of (0.48, 0.42), turns to pure white (0.33, 0.33) at 5.5 V, and becomes bluish white (0.28, 0.29) at 9 V [86].

**Figure 11:**
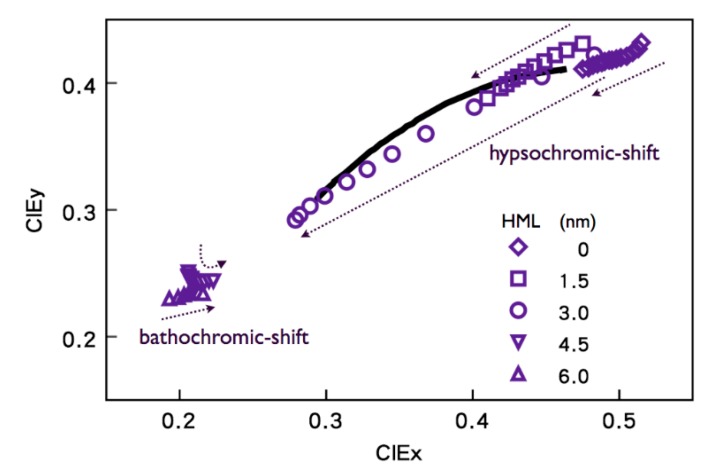
The effect of CML thickness on the device emission tracks, in terms of CIE coordinates (Reproduced from ref. [83]).

As shown in Figure 11, varying the thickness of CML from 0 to 6 nm, the emission gradually changes from the red-emission region to the blue-emission dominated region. Its corresponding chromaticity span gradually increases as the thickness increases, and achieves a maximum at 3 nm. Moreover, as much as the CML thickness increased from 3 to 4.5 or 6 nm, the color coordinates showed a strong blue shift. At 3 nm, the entire emission span is sufficiently wide enough to cover that of daylight, and its emission track matches most closely with the daylight locus. In addition, there exists a chromic-shift turning-point in the vicinity of 4.5 nm, below which all the emissions show a hypsochromic shift, while there is a bathochromic shift above that level. At 4.5 nm, the device shows emission straying around hypsochromic and bathochromic shift at varying voltages [83].

## 6. Effect of CML on CT Tuning

The color temperature of light plays a crucial role in human physiology and psychology [123,124,125,126,127,128,129,130]. Numerous medical studies reported that white light sources with an intense blue emission (5000–6000 K) may cause some serious health issues, such as irreversible retinal damage, physiological disorders, and increasing risk of various cancers resulting from the suppression of melatonin secretion [131]. High color temperature white light stimulates the secretion of cortisol, a hormone that keeps people awake and active [126]. In contrast, a low color temperature light mildly suppresses the nocturnal secretion of oncostatic melatonin, which would help people relax after dusk and sleep well at night [127,128,130]. Devising new lighting sources with a very low color temperature to minimize melatonin suppression is hence no less urgent or less significant than realizing an even higher lighting efficiency. Low color temperature OLEDs can be realized by maximizing the long wavelength (red) emission and minimizing the short wavelength (blue) emission counterpart. Lately, it has been confirmed that an engineered nano CML played a crucial role in regulating the charge carrier (holes and electrons) injection [53]. Jou’s group reported a candlelight-style OLED device with a color temperature of 1920 K by using a TPBi nano CML [47]. Jou’s group also reported an OLED device with a color temperature of 1880 K and a power efficiency of 36 lm·W^−1^ (current efficiency of 54 cd·A^−1^), at 100 cd·m^−2^, by employing a 3-nm BSB:Spiro-2CBP (2:1) blend CML between the blue and orange red emissive layers [53]. Most recently, our group also reported a candlelight emission with fluorescent tube efficacy and color temperature of 2279 K at 100 cd·m^−2^ [132].

Moreover, it has also been found that the nano CML played a crucial role in realizing the tunable color temperature [84,85,86]. Devising a light source with tunable color temperature hence becomes imperative in order to obtain high-quality lighting. In 2009, Jou’s group invented the first sunlight-style color temperature-tunable OLED device by employing a 3 nm TPBi as CML. The resultant sunlight-style OLED device exhibited a power efficiency of 7.0 lm·W^−1^ (at 100 cd·m^−2^) with a color temperature ranging from 2300 and 9700 K (Figure 10b) [86]. Subsequently, Jou’s group further enhanced the performance of the sunlight-style OLED device by using electro-phosphorescent emitters and double nano CMLs. The resultant device showed a power efficiency of 30 lm·W^−1^ with color temperature ranges from 1900 to 3100 K [83].

**Figure 12:**
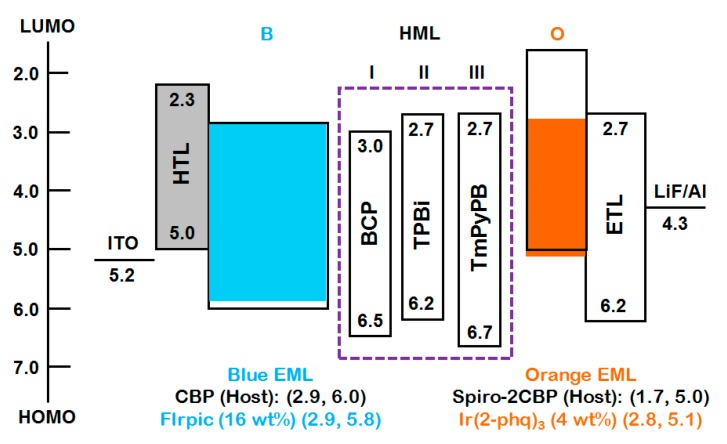
Energy level diagram of the studied devices with different CMLs (Reproduced from ref. [85]).

**Figure 13:**
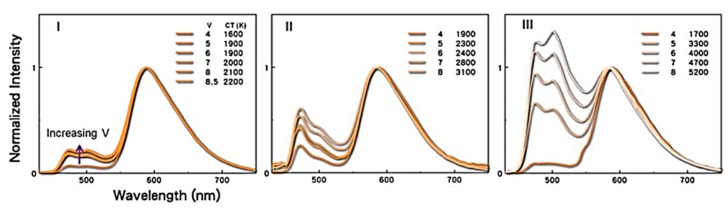
EL spectra comparison of devices with BCP (**I**); TPBi (**II**); and TmPyPB (**III**) CMLs (Reproduced from ref. [85]).

As shown in Figure 12, three electron-transporting materials, BCP, TPBi, and TmPyPB, were investigated as CMLs in OLED devices to realize the large color temperature span. When TmPbPB is employed as a nano CML, the resultant OLED device exhibited the largest color temperature span of 3700 K. This may be attributed to the fact that TmPyPB possessed the highest hole-injection barrier (0.5 eV) and triplet energy (2.8 eV), effectively modulating the flux of holes and confining the triplet excitons within the blue emissive layer. In addition, TmPyPB also showed relatively higher electron mobility than the BCP and TPBi counterparts, leading more excitons to be generated on the blue emissive layer as the operation voltage increased from 4 to 8 V (Figure 13).

**Figure 14:**
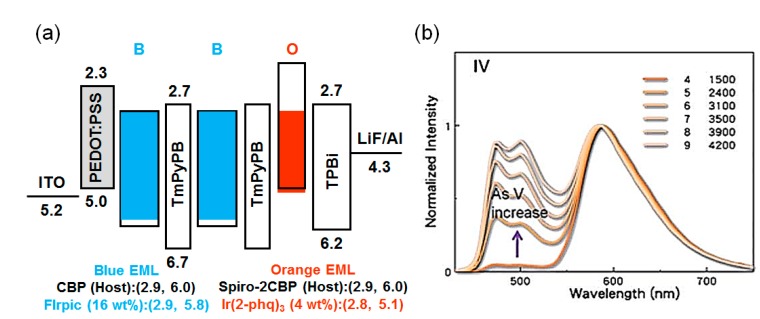
(**a**) Energy-level diagram of Device IV incorporating double CMLs; (**b**) the resulting EL-spectra of double CML device (Reproduced from ref. [85]).

As shown in Figure 14, the color temperature of device with TmPyPB-based nano CML can further reduce to 1500 K by introducing an additional TmPyPB between two sky-blue emissive layers. The resultant double nano CML-based device exhibited a power efficiency of 25 lm·W^−1^ with a color temperature ranging from 1500 to 4200 K as operation voltage increased from 4 to 9 V [85].

Most recently, Jou’s group also reported an OLED device with tunable color temperature from 1580 to 2600 K by using a TPBi CML. The resultant device exhibited the color temperature between dusk-hue (2500 K) and candlelight (1900 K). The color temperature of the OLED device is further tuned from 5200 to 2360 K, which covers daylight-like illumination, by simply adjusting the emissive layer thickness ratio [84].

Unlike numerous favorable functions, a CML may also possess some limitations such as driving voltage enhancement and fabrication complexity in OLED devices. For example, the driving voltage of the sunlight-style OLED device increased from 4.3 to 4.5 as CML thickness increased from 3 to 5 nm [121]. Jou’s group observed that the driving voltage increased from 4.1 to 4.7 V as the thickness of BSB:Spiro-2CBP blend CML increased from 3 to 6 nm [53]. In contrast, host-free deep-blue and chromaticity-tunable OLED devices had not displayed any considerable enhancement in driving voltages when the hole-transporting TAPC and bipolar Spiro-2CBP materials are used as CML, respectively [41,84]. Hence, the driving voltage enhancement can be successfully controlled by using an optimized thickness of suitable carrier modulation material.

## 7. Conclusions

In this article, we have reviewed the comprehensive features of CMLs’ incorporation onto the performance of OLEDs. Incorporation of a nano CML within the emissive layer may enhance aspects of the device performance such as efficiency, maximum luminance, lifetime, chromaticity tenability, very low color temperature, and CT tunability with high or very high CRI and SRI. Nano CML improves the distribution of the entering charge carriers into the available recombination zones and balances the carrier injection. The performance of a nano CML containing numerous types of monochromatic, cold/warm white, sunlight-style, and candlelight-style OLED devices varied with the variation of thickness, incorporation position, and composition.
